# Opposing white matter microstructure abnormalities in 22q11.2 deletion and duplication carriers

**DOI:** 10.1038/s41398-021-01703-1

**Published:** 2021-11-10

**Authors:** Johanna Seitz-Holland, Monica Lyons, Leila Kushan, Amy Lin, Julio E. Villalon-Reina, Kang Ik Kevin Cho, Fan Zhang, Tashrif Billah, Sylvain Bouix, Marek Kubicki, Carrie E. Bearden, Ofer Pasternak

**Affiliations:** 1 Department of Psychiatry, Brigham and Women’s Hospital, Harvard Medical School, Boston, 02115 MA USA; 2 Department of Psychiatry and Biobehavioral Sciences, Semel Institute for Neuroscience and Human Behavior, University of California at Los Angeles, Los Angeles, 90095 CA USA; 3 Department of Psychiatry, Massachusetts General Hospital, Harvard Medical School, Boston, 02114 MA USA; 4 Department of Radiology, Brigham and Women’s Hospital, Harvard Medical School, Boston, 02115 MA USA; 5 Department of Psychology, University of California at Los Angeles, Los Angeles, 90095 CA USA

**Keywords:** Neuroscience, Diseases

## Abstract

Deletions and duplications at the 22q11.2 locus are associated with significant neurodevelopmental and psychiatric morbidity. Previous diffusion-weighted magnetic resonance imaging (MRI) studies in 22q11.2 deletion carriers (22q-del) found nonspecific white matter (WM) abnormalities, characterized by higher fractional anisotropy. Here, utilizing novel imaging and processing methods that allow separation of signal contribution from different tissue properties, we investigate whether higher anisotropy is driven by (1) extracellular changes, (2) selective degeneration of secondary fibers, or (3) volumetric differences. We further, for the first time, investigate WM microstructure in 22q11.2 duplication carriers (22q-dup). Multi-shell diffusion-weighted images were acquired from 26 22q-del, 19 22q-dup, and 18 healthy individuals (HC). Images were fitted with the free-water model to estimate anisotropy following extracellular free-water elimination and with the novel BedpostX model to estimate fractional volumes of primary and secondary fiber populations. Outcome measures were compared between groups, with and without correction for WM and cerebrospinal fluid (CSF) volumes. In 22q-del, anisotropy following free-water elimination remained significantly higher compared with controls. BedpostX did not identify selective secondary fiber degeneration. Higher anisotropy diminished when correcting for the higher CSF and lower WM volumes. In contrast, 22q-dup had lower anisotropy and greater extracellular space than HC, not influenced by macrostructural volumes. Our findings demonstrate opposing effects of reciprocal 22q11.2 copy-number variation on WM, which may arise from distinct pathologies. In 22q-del, microstructural abnormalities may be secondary to enlarged CSF space and more densely packed WM. In 22q-dup, we see evidence for demyelination similar to what is commonly observed in neuropsychiatric disorders.

## Introduction

Copy number variation (CNV) at the 22q11.2 locus is associated with significant neurodevelopmental and psychiatric morbidity. Deletion of the 22q11.2 locus occurs in approximately one in 4000 live births [1]. Medical comorbidities commonly include cardiac defects [2], craniofacial anomalies [3], and immune dysregulation [4]. 22q11.2 deletion carriers (22q-del), furthermore, have elevated risk for autism spectrum disorder, attention deficit-hyperactivity disorder (ADHD), anxiety, intellectual disability, and up to 25% develop psychosis [5, 6]. Duplication of the 22q11.2 locus is more common than the reciprocal deletion (estimated at one out of 700 live births) [7] but was more recently identified and is less well-characterized [8]. The 22q11.2 duplication is associated with incomplete penetrance and variable expressivity, even within the same family; associated features include hypotonia, facial dysmorphia, and developmental delay, although many 22q11.2 duplication carriers (22q-dup) have a normal or near-normal phenotype [9–11]. However, 22q-dup can suffer from a range of comorbidities that partially overlap those reported for 22q-del, including increased risk for autism, ADHD, and mild intellectual disability [12, 13]. In contrast, the 22q11.2 duplication is not associated with an elevated risk of developing psychosis [14], with some studies suggesting that the risk for psychosis in 22q-dup is lower than in the general population [15].

Given the importance of white matter (WM) microstructure for cognitive functioning [16], its disruption in numerous psychiatric and neurological disorders (including psychosis, mood disorders, obsessive-compulsive spectrum disorders, epilepsy, dementia, and Parkinson’s disease) [17–24], and postmortem findings that implicate WM abnormalities in 22q-del [25, 26], there is a growing interest in characterizing the nature of WM microstructural anomalies in 22q-del [27, 28]. Studies to date have applied diffusion-weighted magnetic resonance imaging (MRI), which quantifies the directionality and magnitude of water diffusion in the brain, reflecting the geometrical properties of the environment in which water is diffusing [29, 30]. The fractional anisotropy (FA) index, derived from the diffusion tensor imaging (DTI) model, is the most used index to quantify diffusion properties.

Lower FA has been implicated in multiple idiopathic psychiatric disorders (i.e., psychosis, depression, post-traumatic stress disorder, and obsessive–compulsive spectrum disorders), as well as neurodegenerative disorders, and is often interpreted as a proxy for abnormal WM organization [31, 32]. However, while FA is a sensitive marker to distinguish groups, it is relatively non-specific and influenced by various cellular (e.g., demyelination, axonal degeneration, and cytoskeletal damage) and extracellular (e.g., atrophy and edema) pathologies [31, 33]. Thus, the neurobiological underpinnings of altered FA are unclear.

While there are some earlier studies that reported decreased FA in single WM tracts in 22q-del [27, 34, 35], most diffusion-weighted MRI studies have reported a widespread increase of FA in 22q-del [28, 36–40]. Importantly a recent multi-site DTI study comparing 22q-del with matched healthy individuals (HC) confirmed the finding of *higher* FA in most tracts in 22q-del [41]. Studies investigating WM microstructure in 22q-dup, however, have not yet been conducted. Only one previous MRI study compared gray matter (GM) structure in reciprocal 22q11.2 CNVs. The authors discovered global opposing effects of gene dosage on cortical thickness and surface area, involving widespread reductions in cortical surface area in 22q-del and increases in 22q-dup relative to controls, with the opposite pattern for cortical thickness [42].

In the present study, we compared diffusion MRI data between reciprocal 22q11.2 CNVs and tested whether opposing effects of gene dosage, similar to the effects reported for GM, also occur in WM. To determine the potential mechanisms that might drive FA alterations, we applied advanced dMRI analysis approaches which allowed considering: (1) extracellular and cellular changes, as quantified by free-water (FW) imaging [43]; (2) the role of selective degeneration of crossing fibers, as quantified by the BedpostX model [44, 45]; and (3) the interaction between microstructural diffusion measurements and macrostructural volume changes.

## Methods

### Participants

The sample consisted of 26 22q-del, 19 22q-dup, and 18 HC that passed visual quality control. Patients have been recruited from either (1) Clinical Genetics, Allergy/Immunology, or Craniofacial Clinics from medical centers in the Southern California area, or (2) through local support groups and websites. HC were recruited from the community via web-based advertisements and local schools, pediatric clinics, and community sites. Exclusion criteria for all participants were significant neurological or medical conditions (unrelated to 22q11.2 deletion/duplication), history of head injury with loss of consciousness, insufficient fluency in English, and/or substance abuse or dependency within the past six months. Further exclusion criteria for HC were current or past major mental disorders (except for ADHD or past episodes of depression) and/or intellectual disability (IQ below 70). HC were screened for mental disorders via the Structured Clinical Interview for the Diagnostic and Statistical Manual of Mental Disorders Version 4 [46] or Computerized Diagnostic Interview for Children.

All participants or parents, if participants were under the age of 18, provided written informed consent, and the University of California at Los Angeles Institutional Review Board approved all study procedures.

### Cognitive and clinical assessment

Participants underwent a comprehensive clinical and cognitive test battery administered by trained clinical psychology students and supervised by PhD-level clinicians.

Given the previously reported high rates of neuropsychiatric disorders in 22q-del and 22q-dup [11, 12, 47–49], we collected extensive clinical and cognitive data for the present study. We utilized the Structured Clinical Interview for DSM V (SCID-I) and the Brief Psychiatric Rating Scale- Expanded Version (BPRS) [50–52] to assess neuropsychiatric diagnosis and symptoms. In addition, the Structured Interview for Psychosis-Risk Syndromes (SIPS) [53] was administered to assess for psychotic and prodromal (i.e., psychosis-risk) symptoms. Previous research has suggested that many individuals with the 22q-del present with prodromal symptoms [54] and up to 25% develop psychosis [55, 56, 6]. 22q-dup, on the other hand, does not have an elevated risk for psychosis [14], and in fact, there is some evidence suggesting a lower risk for psychosis than in the general population [15].

Furthermore, we were interested in characterizing global and cognitive functioning. Functional status was, therefore, determined by the Global Functioning Scales (GAF) [57]. In addition, the Wechsler Abbreviation Scale for Intelligence-2 Vocabulary (VOC) and Matrix Reasoning (Matrix) subtests and the Wide Range Achievement Test 4 (WRAT) Reading subtest were used to assessing intelligent quotient (IQ) and reading ability, respectively [58, 59].

### Image acquisition and preprocessing

Images were acquired on a 3 T whole-body scanner (Siemens Magnetom Prisma) with a 32-channel head coil. Scanning protocols were derived from the Lifespan Human Connectome Project (HCP) study, including multi-shell diffusion-weighted images that were acquired with the following protocol: AP and PA sequence each with voxel size = 2.0 × 2.0 × 2.0 mm, TR = 8000 ms, TE = 66 ms, flip angle = 90°, FOV = 208 mm, slice thickness = 2.00 mm, slices = 72, and 108 volumes, including 46 gradient directions with *b* = 1500 s/mm^2^, 46 gradient directions with *b* = 3000 s/mm^2^, 3 gradient directions with *b* = 200 s/mm^2^, 6 gradient directions with *b* = 500 s/mm^2^, and 7 volumes with *b* = 0 s/mm^2^.

All data underwent a visual quality check for movement artifacts, echo-planar imaging (EPI) distortions, and structural abnormalities by investigators blinded to group allocation. We excluded individuals who did not have complete data for the AP and PA sequence and individuals who presented with severe motion artifacts (excluding a total of 25 individuals: 5 HC, 12 22q-del, and 8 22q-dup). We preprocessed the remaining images utilizing the HCP Minimal Preprocessing Pipeline v4.0.0 [60]. First, we applied intensity normalization of the mean b0 image across the diffusion series and utilized b0 pairs to estimate and correct EPI distortion, using Topup, FSL [61]. Next, diffusion images were corrected for eddy-current induced field inhomogeneities, head motion, and gradient nonlinearity utilizing FSL. Last, we used structural images to mask diffusion images [62].

### Image analyses

#### FA Analyses

We first investigated group differences in FA to determine the comparability of our findings to previous findings of increased FA in 22q-del. From the preprocessed diffusion-weighted images, we, therefore, computed diffusion tensors utilizing a least-squares fit and calculated FA maps from these tensors [63].

#### Cellular and extracellular analyses

To identify if the FA abnormalities originate from the cellular or extracellular domains, we fitted a two-compartment FW model to the multi-shell diffusion data using a regularized non-linear fit [43]. One compartment models signals from water molecules that diffuse unhindered in the extracellular space using an isotropic diffusion tensor with a fixed diffusivity of FW in body temperature. The fractional volume of this FW compartment is identified as FW. Previous studies have demonstrated that an increase of FW indicates extracellular pathological processes such as atrophy, edema, and neuroinflammation [64]. The second compartment models hindered and restricted diffusion of water molecules in the vicinity of the cellular space using a diffusion tensor, from which FA of the tissue (FA_T_) is derived [43, 64, 65]. FA_T_ represents anisotropy following the elimination of FW contribution, and therefore FA_T_ more specifically reflects changes in the WM tissue (such as changes in myelination and axonal membrane health) than FA [43, 66]. FW imaging studies in healthy aging and in several disorders (including psychosis [67, 68], mood disorders [69, 70], eating disorders [71], traumatic brain injury [72], dementia [73, 74], and Parkinson’s disease [75, 76]) have demonstrated the importance of separating cellular and extracellular WM abnormalities [77].

#### Identification of secondary degeneration

We used the crossing fiber BedpostX model [44] to obtain information about the complex fiber architecture at each voxel. Previous studies have suggested that increased FA could result from selective degeneration of secondary fibers in regions with crossing fibers [78, 79]. This model considers three fiber populations with distinct orientations and an additional isotropic compartment. The model parameters are estimated in each voxel using a Bayesian framework and include the volume fractions for the primary (F1), secondary (F2), and tertiary (F3) fiber populations, as well as the fractional volume of the isotropic compartment (F_iso_). Degeneration of secondary fibers is expected to be indicated by a decrease of F2 but not F1. Previous studies have demonstrated that this selective degeneration of secondary fibers yields less partial volume in crossing fibers areas, resulting in an increase of FA values [78].

#### Tract-based spatial statistics

To facilitate group comparisons, we employed the tract-based spatial statistics (TBSS) pipeline [80–82] by nonlinear registration of each subject’s FA image to the FA target image and skeleton that was created by the Enhanced Neuroimaging Genetics by Meta-Analysis (ENIGMA) DTI Working Group. We used an in-house script (https://pnlbwh.github.io/TBSS/TUTORIAL.html) that performed all registrations using the Advanced Normalization Tools (ANTs) toolbox [83, 84]. FA, FA_T_, and FW maps were projected onto the ENIGMA skeleton [85] to perform statistical analyses.

On the output of the BedpostX model, we applied FSL’s tbss_x script. This script provides intra- and inter-subject reassignment of fiber fractions, thus ensuring that a fiber population in one subject corresponds to the same fiber population across all individuals [45]. We projected F1, F2, and F_iso_ maps onto the skeleton provided by ENIGMA to perform statistical analyses. (F3 was not included in the analyses due to its inherently low and inconsistent values [86]).

#### Accounting for macrostructural volumetric measures

To test if macrostructural volume measures interact with the microstructural measures, we utilized a novel segmentation algorithm [87] that allowed for the segmentation of the diffusion image into CSF, WM, GM, and total intracranial volume (ICV, sum of GM + WM + CSF). Following this, we calculated normalized measurements by dividing CSF, WM, and GM volumes by ICV. We used these normalized volumes for all statistical analyses.

### Statistical analyses

For all TBSS analyses, non-parametric voxel-wise permutation tests for each voxel on the WM skeleton were performed in FSL’s Randomize [81]. We tested data against a null distribution generated with 5000 permutations for each contrast using threshold-free cluster enhancement [82] and family wise error correction at a significance level of *p* < 0.05 and included age, sex, and motion as covariates.

For each of the maps (FA, FA_T_, FW, F1, F2, and F_iso_), we conducted voxel-wise *F*-tests and post hoc *t*-tests to compare the three groups (22q-del, 22q-dup, and HC).

We performed all subsequent analyses using SPSS Version 26 and GraphPad Prism 8.2.0. First, we correlated FA_T_ and FW values with IQ [58, 59], global functioning [57], and the positive and negative symptom scales measured with the Structured Interview for Prodromal Syndromes (SIPS) [53] for 22q-del and 22q-dup separately. A *p*-value < .013 was considered significant (Bonferroni corrected for four tests).

Next, to investigate group differences in macrostructural volume, we carried out four ANCOVAs with ICV, CSF, WM, or GM volume as the independent variable, group as the dependent variable (22q-del, 22q-dup, and HC), and age, sex, and motion as covariates. A *p*-value < 0.013 was considered significant (Bonferroni corrected for four tests). In the case of a significant group effect in the ANCOVA, we then calculated post hoc *t*-tests between the single groups (*p*-values were again Bonferroni corrected).

Last, we included the volumes that demonstrated group differences in our previous analyses to identify the effect of macrostructural volume on microstructure. We, therefore, repeated the voxel-wise *F*-test and post-hoc t-tests comparing FA_T_, FW, F1, F2, and F_iso_, between groups with the volumes that were different between groups as an additional covariate.

## Results

### Demographics

Demographic information is shown in Table 1. The three groups did not differ in terms of age or sex. 22q-del and 22q-dup had poorer cognitive performance and general functioning than HC, with impairments being more pronounced for 22q-del. In addition, both groups reported more psychiatric symptoms than HC. As displayed in Table 2, the most frequent DSM-V diagnoses in 22q-del and 22q-dup were anxiety disorders, autism spectrum disorder, ADHD, and mood disorders. As described previously [6, 55, 56], 22q-del reported more prodromal psychotic symptoms than 22q-dup and HC. However, only four 22q-del were diagnosed with overt psychotic disorder.

**Table 1:** Demographics and clinical data.

	Healthy controls (HC)	22q11.2 deletion carriers (22q-del)	22q11.2 duplication carriers (22q-dup)	Test statistic
*N*	18	26	19	
Age (years): [mean ± std]	[19.51 ± 10.91]	[20.47 ± 8.80]	[22.24 ± 14.41]	*F* = 0.28, d*f* = 2, *p* = 0.76
Sex, F = female / M = male: n (%)	F: 14 (78%), M: 4 (22%)	F: 17 (65%), M: 9 (35%)	F: 8 (42%), M: 11 (58%)	*H* = 5.13, d*f* = 2, *p* = 0.077
VOC, T-score: *n* [mean ± std]	18 [60.06 ± 9.60]	26 [41.77 ± 8.42]	19 [46.58 ± 11.37]	*F* = 19.34, d*f* = 2, *p* < 0.001
Matrix, T-score: *n* [mean ± std]	18 [58.22 ± 9.48]	26 [40.00 ± 9.52]	19 [48.42 ± 11.66]	*F* = 17.04, d*f* = 2, *p* < 0.001
Full Scale IQ: *n* [mean ± std]	18 [115.78 ± 15.26]	26 [84.35 ± 12.40]	19 [95.74 ± 18.36]	*F* = 22.79, d*f* = 2, *p* < 0.001
WRAT4, T-score: *n* [mean ± std]	15 [59.67 ± 11.33]	26 [45.38 ± 6.26]	19 [45.21 ± 14.07]	*F* = 10.42, d*f* = 2, *p* < 0.001
GAF: *n* [mean ± std]	18 [85.93 ± 12.45]	26 [54.71 ± 18.30]	19 [59.65 ± 13.15]	*F* = 23.75, d*f* = 2, *p* < 0.001
BPRS Total Score: *n* [mean ± std]	17 [24.76 ± 1.60]	25 [36.80 ± 9.54]	17 [33.12 ± 6.93]	*F* = 13.86, d*f* = 2, *p* < 0.001
SIPS Positive Symptoms total score: *n* [mean ± std]	16 [1.13 ± 2.31]	26 [4.50 ± 5.96]	18 [2.44 ± 2.97]	*F* = 3.06, d*f* = 2, *p* = 0.054
SIPS Negative Symptoms total score: *n* [mean ± std]	16 [0.94 ± 1.91]	26 [9.77 ± 8.04]	18 [6.61 ± 5.41]	*F* = 10.15, d*f* = 2, *p* < 0.001
SIPS Disorganized Symptoms total score: *n* [mean ± std]	16 [.63 ± 1.15]	26 [4.54 ± 3.70]	17 [2.47 ± 3.12]	*F* = 8.41, d*f* = 2, *p* < 0.001
Meets criteria for psychosis-risk symptoms	1	10	3	
• The Wechsler Abbreviation Scale for Intelligence-2 Vocabulary (VOC) and Matrix Reasoning (Matrix) subtests and the Wide Range Achievement Test 4 (WRAT4) Reading subtest were used to assessing IQ and reading ability, respectively [58, 59]. Higher values present better performance. • The Global Assessment of Functioning score (GAF) was employed to assess general functioning. Lower values present more functional impairments, with values below 70 being associated with at least moderate symptoms [57]. • Several rating scales were administered to determine psychiatric symptoms:o BPRS = Brief Psychiatric Rating Scale- Expanded Version: 24 items: 1 (not present), 2 (very mild), 3 (mild), 4 (moderate), 5 (moderately severe), 6 (severe), 7 (extremely severe) [50–52]o SIPS = Structured Interview for Psychosis Risk Syndromes [53]: symptoms are rated on the following scale: 0 (absent), 1 (questionably present), 2 (mild), 3 (moderate), 4 (moderately severe), 5 (severe but not psychotic), 6 (severe and psychotic).

**Table 2:** Psychiatric diagnoses in CNV carriers*.

	22q11.2 deletion carriers (22q-del) (%)	22q11.2 duplication carriers (22q-dup) (%)
Substance-related disorders	3.85	0
Schizophrenia and other psychotic disorders	11.54	0
Mood disorders	26.92	26.32
Anxiety disorders	61.54	42.11
Obsessive–compulsive spectrum disorder	7.69	0
Eating disorders	3.85	0
Autism Spectrum Disorder	34.62	31.58
Attention deficit hyperactivity disorder	46.15	52.63

*Obtained from the Structural Clinical Interview for DSM V (SCID-I)

### FA analyses

**Fig. 1:**
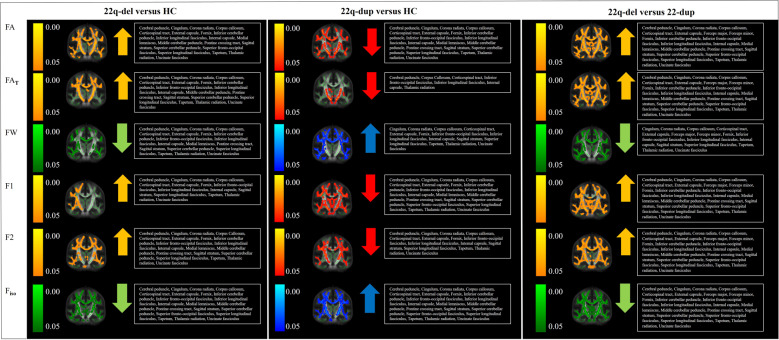
Group comparisons between 22q11.2 deletion carriers (22q-del) and healthy controls (HC) (left), 22q11.2 duplication carriers (22q-dup) and HC (middle), and 22q-del and 22q-dup. Figure 1 displays the results from Tract-Based Spatial Statistics and Randomize [81, 82]. The white matter skeleton (thresholded at fractional anisotropy (FA) > 0.25) is shown in green on top of the average image of all registered FA images. Voxels that demonstrated significant group differences are thickened to increase visibility. The 22q-del group compared with the HC group (left) showed higher fractional anisotropy (FA; significant regions highlighted in orange), which remained higher in FA of the tissue (FA_T_) values (in orange) after accounting for the effect of extracellular free-water (FW). 22q-del also showed lower FW values than HC (in green) and higher fractions of both the primary (F1) and secondary (F2) fiber populations (in orange), and lower fractional volume of the isotropic compartment F_iso_ (in green). In contrast, 22q-dup compared with HC (middle) presented with lower FA values (in red), as well as lower FA_T_ (in red), higher FW values (in blue), lower fractions of the primary and secondary fiber populations (F1, F2, in red) and higher F_iso_ (in blue). A direct comparison between 22q-del and 22q-dup is provided on the right, showing widespread group differences. 22q-del displayed higher FA/FA_T_/F1/F2 (in orange) and lower FW/F_iso_ (in green). Please note that TBSS provides a voxel-wise rather than tract-specific output. The voxels that demonstrate group differences are widespread and located in most of the main fiber tracts. For a tract-by-tract comparison of FA_T_ and FW between groups, please see Supplementary Table 1.

An *F*-test revealed a significant group effect on FA in 54% of the WM skeleton. Post hoc *t* tests demonstrated that 22q-del had significantly *higher* FA than HC in 41% of the WM skeleton, and 22q-dup had significantly *lower* FA than HC in 37% of the WM skeleton. Further, 22q-del displayed significantly *higher* FA than 22q-dup in 66% of the WM skeleton (Fig. 1).

### Cellular and extracellular analyses

F-tests comparing the output of the FW imaging measures revealed significant group effects on FA_T_ in 45% of the WM skeleton and on FW in 72% of the WM skeleton. Post hoc *t* tests revealed that relative to HC, 22q-del had *higher* FA_T_ (in 24% of the WM skeleton) and *lower* FW (in 49% of the WM skeleton). 22q-dup presented with the opposite pattern: *lower* FA_T_ (in 8% of the WM skeleton) and *higher* FW (in 59% of the WM skeleton) than HC. In addition, 22q-del demonstrated *higher* FA_T_ (54% of the WM skeleton) and lower FW (76% of the WM skeleton) than 22q-dup (Fig. 1, Table 3).

**Table 3:** FA_T_ and FW group comparisons *.

	Covariates: age, sex, motion	Covariates: age, sex, motion, CSF volume	Covariates: age, sex, motion, WM volume
*FA*_*T*_			
F-test	**45% of the WM skeleton**	**24% of the WM skeleton**	**27% of the WM skeleton**
22q-del versus HC	22q-del > HC: 24%	22q-del > HC: –	22q-del > HC: 1,5%
	22q-del < HC: –	22q-del < HC: –	22q-del < HC: –
22q-dup versus HC	22q-dup > HC: –	22q-dup > HC: –	22q-dup > HC: –
	22q-dup < HC: 8%	22q-dup < HC: 23%	22q-dup < HC: 7%
22q-del versus 22q-dup	22q-del > 22q-dup: 54%	22q-del > 22q-dup: 40%	22q-del > 22q-dup: 35%
	22q-del < 22q-dup: –	22q-del < 22q-dup: –	22q-del < 22q-dup: –
*FW*			
F-test	**72% of the WM skeleton**	**63% of the WM skeleton**	**55% of the WM skeleton**
22q-del versus HC	22q-del > HC: –	22q-del > HC: –	22q-del > HC: –
	22q-del < HC: 49%	22q-del < HC: 3%	22q-del < HC: –
22q-dup versus HC	22q-dup > HC: 59%	22q-dup > HC: 60%	22q-dup > HC: 60%
	22q-dup < HC: –	22q-dup < HC: –	22q-dup < HC: –
22q-del versus 22q-dup	22q-del > 22q-dup: –	22q-del > 22q-dup: –	22q-del > 22q-dup: –
	22q-del < 22q-dup: 76%	22q-del < 22q-dup: 69%	22q-del < 22q-dup: 60%

*CSF* cerebrospinal fluid, *FA*_*T*_ fractional anisotropy of cellular tissue, *FW* extracellular free-water, *HC* healthy controls, *WM* white matter, *22q-del* 22q11.2 deletion carriers, *22q-dup* 22q11.2 duplication carriers.

*All group comparisons were conducted utilizing non-parametric voxel-wise permutation tests for each voxel on the WM skeleton in FSL’s Randomize [81]. We tested data against a null distribution generated with 5000 permutations for each contrast using threshold-free cluster enhancement [82] and family wise error correction at a significance level of *p* < 0.05.

We did not observe a significant correlation between FA_T_ or FW and IQ [58, 59], global functioning [57], nor the SIPS positive and negative symptom scales in either 22q-del or 22q-dup carriers.

### Complex fiber architecture and the identification of selective degeneration

The *F*-tests comparing the BedpostX fractional volume measures between groups showed a significant group effect on the fraction of both primary (F1 in 36% of the WM skeleton) and secondary (F2 in 40% of the WM skeleton) fibers, as well as the partial volume fraction of the isotropic compartment (F_iso_ in 66% of the WM skeleton). Post-hoc t-tests revealed that 22q-del had *higher* primary (F1 in 8% of the WM skeleton) and secondary (F2 in 28% of the WM skeleton) fiber fractions and a *lower* isotropic fraction (F_iso_ in 49% of the WM skeleton) than HC, which is not consistent with a pattern of degeneration of secondary fibers [78, 79]. Similar to the FA results, 22q-dup presented with the opposite pattern: *lower* primary (26% of the WM skeleton) and secondary (21% of the WM skeleton) fiber fractions than HC and a *higher* isotropic fraction (in 48% of the WM skeleton) than HC (Fig. 1), which also is not consistent with a pattern of degeneration of secondary fibers [78, 79]. For group comparisons between 22q-del and 22q-dup, please see Table 3.

### Comparison of macrostructural volumetric measures

**Fig. 2:**
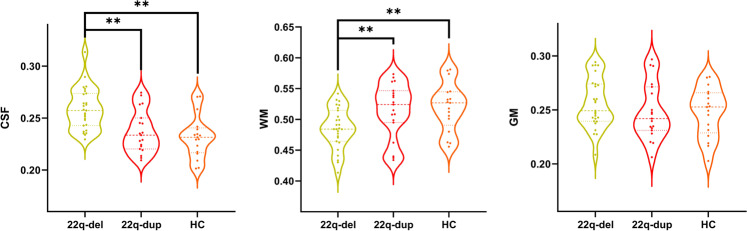
Macrostructural volumetric measures across groups. Group comparison for relative cerebrospinal fluid volume (CSF), relative white matter (WM) volume, and relative gray matter (GM) volume between 22q11.2 deletion carriers (22q-del), 22q11.2 duplication carriers (22q-dup), and healthy controls (HC). 22q-del had significantly higher CSF volume and lower WM volume relative to 22q-dup and HC. GM volume did not significantly differ between groups.

We did not observe a significant overall group effect on ICV (*F* = 3.75, d*f*1 = 2, d*f*2 = 58, *p* = 0.029) or GM volume (*F* = 2.07, d*f*1 = 2, d*f*2 = 58, *p* = 0.14). However, there was a significant group effect on WM volume (*F* = 17.26, d*f*1 = 2, d*f*2 = 58, *p* < 0.001) and CSF volume (*F* = 28.54, d*f*1 = 2, d*f*2 = 58, *p* < 0.001). Post hoc *t*-tests revealed that 22q-del had *lower* WM volume than both 22q-dup (*p* = 0.062) and HC (*p* = 0.050). In addition, 22q-del had *higher* CSF volume than both 22q-dup (*p* = 0.002) and HC (*p* < 0.001) (Fig. 2).

### Accounting for macrostructural volumetric measures

**Fig. 3:**
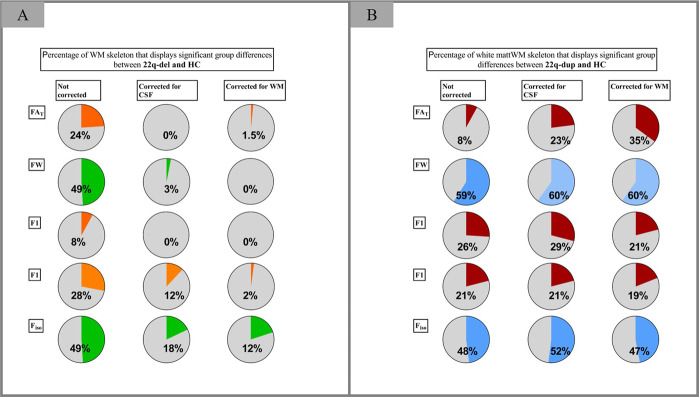
The effect of controlling for macrostructural volume on the percentage of white matter (WM) skeleton that displays significant group differences. Comparing 22q11.2 deletion carriers (22q-del) and healthy controls (HC) (Panel **A**) shows that without controlling for macrostructural volume (left column), 22q-del displays significant differences in all microstructural parameters (FAt, FW, F1, F2, F_iso_). When controlling for relative cerebrospinal fluid (CSF) volume (middle column) or relative WM volume (right column), these group differences disappear or diminish. Comparing 22q11.2 duplication carriers (22q-dup) and HC (Panel **B**) shows that the percentage of skeleton voxels with significant findings (left column) remain similar when controlling for CSF volume (middle row) or WM volume (right row).

Given the group effect on CSF and WM volume, we conducted two additional analyses of the voxel-wise *F*- and *t* tests comparing FA_T_, FW, F1, F2, and F_iso_: once controlling for CSF, and once controlling for WM volume. This was done to test whether the microstructural impairments reported above could be accounted for by volumetric differences between groups. Indeed, when correcting previous analyses for WM volume, differences between 22q-del and HC in FW and F1 were no longer significant, and differences in FA_T_, F2, and F_iso_ were more localized (Table 3, Table 4, and Fig. 3). Similarly, when adjusting previous analyses for CSF volume, differences between 22q-del and HC in FA_T_ and F1 were no longer significant, and differences in FW, F2, and F_iso_ were more localized (Table 3, Table 4, and Fig. 3). Notably, group differences between 22q-dup and HC remained stable after adding CSF or WM volume as covariates (Table 3, Table 4, and Fig. 3).

**Table 4:** F1, F2, and F_iso_ group comparisons*.

	Covariates: age, sex, motion	Covariates: age, sex, motion, CSF volume	Covariates: age, sex, motion, WM volume
*F1*			
*F*-test	**36% of the WM skeleton**	**32% of the WM skeleton**	**14% of the WM skeleton**
22q-del versus HC	22q-del > HC: 8%	22q-del > HC: –	22q-del > HC: –
	22q-del < HC: –	22q-del < HC: –	22q-del < HC: –
22q-dup versus HC	22q-dup > HC: –	22q-dup > HC: –	22q-dup > HC: –
	22q-dup < HC: 26%	22q-dup < HC: 29%	22q-dup < HC: 21%
22q-del versus 22q-dup	22q-del > 22q-dup: 43%	22q-del > 22q-dup: 31%	22q-del > 22q-dup: 21%
	22q-del < 22q-dup: –	22q-del < 22q-dup: –	22q-del < 22q-dup: –
*F2*			
*F*-test	**40% of the WM skeleton**	**26% of the WM skeleton**	**23% of the WM skeleton**
22q-del versus HC	22q-del > HC: 28%	22q-del > HC: 12%	22q-del > HC: 2%
	22q-del < HC: –	22q-del < HC: –	22q-del < HC: –
22q-dup versus HC	22q-dup > HC: –	22q-dup > HC: –	22q-dup > HC: –
	22q-dup < HC: 21%	22q-dup < HC: 21%	22q-dup < HC: 19%
22q-del versus 22q-dup	22q-del > 22q-dup: 46%	22q-del > 22q-dup: 34%	22q-del > 22q-dup: 28%
	22q-del < 22q-dup: –	22q-del < 22q-dup: –	22q-del < 22q-dup: –
*F*_*iso*_			
*F*-test	**66% of the WM skeleton**	**55% of the WM skeleton**	**50% of the WM skeleton**
22q-del versus HC	22q-del > HC: –	22q-del > HC: –	22q-del > HC: –
	22q-del < HC: 49%	22q-del < HC: 18%	22q-del < HC: 12%
22q-dup versus HC	22q-dup > HC: 48%	22q-dup > HC: 52%	22q-dup > HC: 47%
	22q-dup < HC: –	22q-dup < HC: –	22q-dup < HC: –
22q-del versus 22q-dup	22q-del > 22q-dup: –	22q-del > 22q-dup: –	22q-del > 22q-dup: –
	22q-del < 22q-dup: 72%	22q-del < 22q-dup: 64%	22q-del < 22q-dup: 47%

*CSF* cerebrospinal fluid, *F1* fractional volume of primary fiber population, *F2* fractional volume of secondary fiber population, *F*_*iso*_ fractional volume of the isotropic compartment, *HC* healthy controls, *WM* white matter, *22q-del* 22q11.2 deletion carriers, *22q-dup* 22q11.2 duplication carriers.

*All group comparisons were conducted utilizing non-parametric voxel-wise permutation tests for each voxel on the WM skeleton in FSL’s Randomize [81]. We tested data against a null distribution generated with 5000 permutations for each contrast using threshold-free cluster enhancement [82] and family wise error correction at a significance level of *p* < 0.05.

## Discussion

The present study provides novel information regarding WM abnormalities in 22q11.2 CNV carriers. First, using new multi-shell diffusion sequences, we confirmed previous findings of abnormally *high* FA in 22q-del. Further analyses demonstrated that the observed higher FA remains following the elimination of extracellular FW and cannot be attributed to selective degeneration of secondary fibers. However, higher FA, which was accompanied by higher primary and secondary fiber fractions and lower extracellular space, was associated with enlarged CSF and smaller WM volumes in 22q-del. The finding of reduced WM volume and reduced extracellular space within the WM suggests that the WM in 22q-del may be abnormally densely packed.

**Fig. 4:**
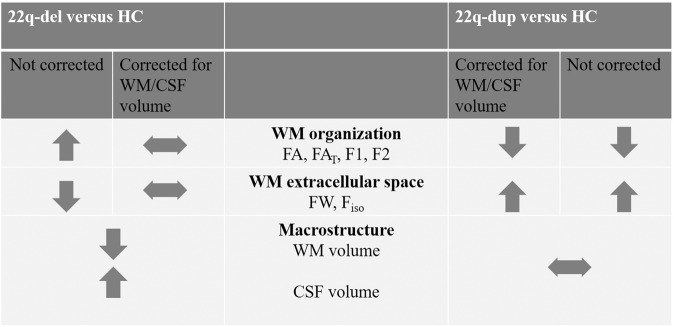
Summary of overall results. When not correcting for macroscopic volumes, 22q11.2 deletion (22q-del) and 22q11.2 duplication carriers (22q-dup) show opposite patterns of white matter (WM) changes compared with healthy controls (HC). Significant macrostructural changes (lower WM/ higher cerebrospinal (CSF) volume) were found in 22q-del but not in 22q-dup. When controlling for macrostructural effects, microstructural changes are no longer significant in 22q-del but remain significant in 22q-dup. Taken together, these results do not support gene dosage as a direct cause for micro- or macrostructural differences in 22q-del and 22q-dup.

Further, we found that 22q-del presents with distinct WM microstructural abnormalities compared to those with 22q-dup. In 22q-dup, we found significantly lower FA and lower primary and secondary fiber fractions, as well as higher extracellular space, relative to controls. This pattern is similar to what has been observed for many behaviorally defined neuropsychiatric disorders, such as psychosis, mood disorders, obsessive-compulsive disorder, and dementia [88–93]. In contrast to 22q-del, the microstructural changes were not associated with volumetric changes, suggesting that distinct pathologies may underlie WM pathology in reciprocal 22q11.2 CNVs (Fig. 4).

### WM architecture in 22q-del

We observed widespread higher FA in 22q-del than HCs, affecting most of the main WM tracts, and no regions with lower FA in 22q del. Previous studies examining FA in 22q-del described inconsistent results. Some smaller studies suggested lower FA in localized tracts in 22q-del compared to HC [94, 27, 34, 95, 35]. However—aligning with our findings—prior studies found widespread increased FA [28, 36, 37], along with decreased extracellular space [96]. Notably, the most extensive multi-site study to date [41] also demonstrated lower diffusivity in 22q-del and higher FA in most fiber tracts. As a possible explanation for the inconsistencies, the authors speculated that FA findings may be age-dependent [39, 40].

To better understand the finding of higher FA in our sample, we examined if it is driven by (1) extracellular changes, (2) selective degeneration of secondary fibers, or (3) macrostructural volumetric effects. Utilizing the FW imaging approach [43, 64] to control for extracellular changes, widespread higher FA_T_ was still observed in 22q-del, suggesting that extracellular changes alone did not explain the increase in FA. We also found lower FW, indicating a smaller extracellular space within WM in 22q-del than HC. Using the BedpostX method, which allows us to differentiate the primary and secondary fiber populations in one voxel, we found a higher proportion of both primary and secondary fibers in 22q-del compared to HC. This pattern is not consistent with selective degeneration of crossing fibers, which was found to cause higher FA in regions with crossing fibers in healthy aging and Alzheimer’s Disease populations [78, 79]. Instead, the BedpostX results in 22q-del aligned with the FW imaging results and showed a higher proportion of cellular volume at the expense of smaller extracellular volume at each voxel. In addition, we observed lower overall WM volumes and increased CSF volumes in 22q-del relative to HC. Importantly, when adjusting our statistical analyses for these macrostructural group differences, the changes in FA and FA_T_ between 22q-del and HC were no longer significant.

While our current analysis cannot deduce causality, we speculate that enlarged CSF volume (i.e., in ventricles and around the brain parenchyma) in 22q-del might contribute to smaller WM volume, which then leads to reduced extracellular space within the WM and therefore more densely packed cellular structures. Several previous studies [97–99] have shown increased CSF in 22q-del, with the most frequent changes observed for the lateral and third ventricle. Further, research utilizing a 22q-del mouse model has reported early and progressive ventricle enlargement due to defective ciliary motility which in turn is caused by elevated dopamine receptor levels [100], suggesting a potential neurobiological mechanism underlying CSF abnormalities in 22q-del. At the same time, postmortem studies in 22q-del have suggested that gliotic and neuronal migration defects might alter WM microstructure and density [25, 26]. We, therefore, acknowledge that future longitudinal studies are needed to determine how micro and macrostructural abnormalities interact with one another in 22q11.2 CNV carriers.

In line with our proposed interpretation of more densely packed WM in 22q-del, other clinical populations that observed higher FA (i.e., infants with extremely preterm birth and individuals with Williams’s syndrome) have speculated that the increased FA could be reflective of greater WM packing density [101–103]. Furthermore, diffusion studies in individuals with idiopathic normal pressure hydrocephalus have reported regionally higher FA and—in line with our hypothesis—a positive correlation between FA and ventricular volume [104]. We speculate that the neurological consequences of more densely packed WM might include reduced efficiency of electric signal transport or less effective extracellular matrix; however, preclinical studies investigating this possibility in 22q-del models have not yet been done. Indeed, previous research in (mild) traumatic brain injury and neurodegenerative disorders has suggested that axonal swellings or beading translate into disturbed axonal conduction and synaptic transmission [105]. However, we acknowledge that our interpretation is speculative, and future studies are needed to explore the underlying neurobiological basis of WM abnormalities in 22q11.2 CNV carriers.

Along the same lines, future longitudinal studies observing macro- and microstructural relationships are needed to explore the role of brain pathologies underlying psychiatric symptoms in 22q-del. Similar to our finding of enlarged CSF in 22q-del, previous studies have highlighted the role of increased CSF in various neurological and psychiatric disorders, including dementia [106] and mood disorder [107], and enlarged ventricles are considered one of the hallmarks of psychosis [108]. However, most previous studies have reported decreased rather than increased FA in neuropsychiatric disorders, such as dementia, mood disorders, and psychosis [17–24]. Interestingly, increased FA has been reported in populations at clinical high risk for psychosis, which might be associated with an altered maturational WM trajectory and an earlier peak of WM development [109]. Even though we found increased FA in 22q-del relative to controls, there are important differences from other clinical high-risk populations. Specifically, subjects with 22q-del present with various other medical and clinical comorbidities, including autism spectrum disorder, intellectual disability, and anxiety disorder. This, together with the observed widespread macro-and microstructural abnormalities suggests that 22q-del is characterized by early neurodevelopmental abnormalities, which may be independent of the later risk to develop psychosis. Future longitudinal studies, including a follow-up of the current cohort, may shed more light on the relationship between psychosis onset in the 22q-del individuals and changes in dMRI measures.

### WM architecture in 22q-dup

As the first study to investigate WM architecture in 22q-dup, our findings indicate that the 22q11.2 duplication is associated with an opposing pattern of WM alterations to those that characterize 22q-del. Specifically, 22q-dup demonstrated lower FA and FA_T_, lower primary and secondary fiber fractions (F1 and F2), and higher isotropic space (FW and *F*_iso_) than HC. Interestingly, while we observed a high spatial overlap between abnormalities in 22q-del and 22q-dup, in 22q-dup, microstructural WM abnormalities were independent of macrostructural volume. Similar WM abnormalities (lower FA/FA_T_, higher FW) occur in healthy aging, in neurodegenerative disorders such as dementia, as well as psychiatric disorders including depression and schizophrenia [70, 74, 78, 110–112].

The opposing WM architecture findings in 22q-del and 22q-dup are consistent with imaging studies of other reciprocal CNVs, including 15q11.2 and 16p11.2, in which opposite effects on WM structure have also been observed [113, 114]. Authors of the cited studies speculate that findings might indicate a dosage effect of genes within the affected locus on brain structure. The interpretation of a 22q11.2 gene dosage effect on WM is particularly compelling, given that several genes essential for myelination [115] and cortical circuit formation [116] are located within the 22q11.2 locus. Specifically, the 22q11.2 region encodes the Nogo-66 receptor gene, a growth inhibitor essential for myelin-depended regulation of plasticity [117]. Indeed, one previous study demonstrated that increased FA in 22q-del was associated with an altered dosage of the Nogo-66 receptor gene. The authors speculated that the variations in the Nogo-66 receptor gene might lead to a lack of myelination inhibition, which translates into higher FA [118].

While future studies are needed to further explore the interaction between genes within the 22q11.2 locus and WM structure, our results suggest that the pathologies underlying opposing patterns of WM abnormalities in 22q-del and 22q-dup might differ from one another. Specifically, while lower FA in 22q-dup could be related to less myelin, the higher FA observed in 22q-del is less likely to result from abnormally *increased* myelination, given previous postmortem studies [25, 26] and the severe functional abnormalities [5, 119] associated with 22q-del. Instead, our findings suggest more complex differences between these reciprocal CNVs, in which microstructural WM changes are related to macrostructural volume changes in 22q11.2 deletion, but not 22q11.2 duplication. Postmortem and animal studies are warranted to determine the underlying neurobiology of the observed effects [120, 121].

### Limitations and future directions

The limited sample size did not allow us to investigate subgroups of 22q11.2 CNV carriers with particular psychiatric phenotypes or to explore the association between brain structure and clinical and cognitive impairments in depth. Future investigations in larger cohorts are warranted to compare 22q-del with and without psychotic symptoms, given the increased risk for psychosis in 22q-del [56]. Specifically, such studies could investigate the relationship of brain structural abnormalities with increased risk for psychosis or other common psychiatric disorders in 22q-del and 22q-dup, such as anxiety, mood, and developmental disorders.

As highlighted above, longitudinal studies are also of interest to establish trajectories of neuroanatomical abnormalities and to further elucidate the complex interaction between microstructural and macrostructural abnormalities. Last, while we applied advanced models to investigate WM pathology in 22q-del and 22q-dup, we could not include all factors that might influence the diffusion signal. For example, we could not account for potential vascular abnormalities, which may be present in 22q11.2 CNV carriers, affecting diffusion MRI measurements [122].

## Conclusion

Here, we conducted the first study to apply advanced dMRI analysis methods to elucidate WM microstructure abnormalities in reciprocal 22q11.2 CNVs. Findings revealed opposing effects of the 22q11.2 deletion and duplication on WM architecture. In 22q-del, we identified an association between WM microstructure findings and macrostructural volumetric measures, suggesting microstructural abnormalities are directly related to macrostructural features. WM alterations in 22q-dup, however, appear similar to those observed in other neuropsychiatric disorders such as schizophrenia, bipolar disorder, and dementia, suggesting reduced myelination of WM fibers. Hence, the pathology underlying these opposing effects may differ between 22q-del and 22q-dup and thus does not suggest a direct gene dosage effect on WM. Further longitudinal and translational studies are needed to elucidate the mechanisms underlying WM microstructure and macrostructure alterations resulting from CNV at the 22q11.2 gene locus.

## Supplementary information

Supplementary Material
