# Supplementary material for: Opposing white matter microstructure abnormalities in 22q11.2 deletion and duplication carriers
Source: Transl Psychiatry. 2021 Nov 10;11:580. doi: 10.1038/s41398-021-01703-1 (PMC8581007; doi:10.1038/s41398-021-01703-1)
Supplement: Supplementary file 1 — Supplementary Material [file 41398_2021_1703_MOESM1_ESM.docx]

| **Table S1:** Tract-wise comparison between healthy individuals, 22q11.2 deletion carriers (22q-del), and 22q11.2 duplication carriers (22q-dup) utilizing ANCOVAS (corrected for age, sex, and motion) | | |
| --- | --- | --- |
|  | **FA_T_** | **FW** |
| Anterior corona radiata left | F=6.40, df=2, p=.003  22q-dup < HC < 22q-del | F=20.34, df=2, p<.001  22q-del <HC <22q-dup |
| Anterior corona radiata right | F=2.88, df=2, p=.064 | F=19.50, df=2, p<.001  22q-del <HC <22q-dup |
| Anterior limb of internal capsule left | F=23.16, df=2, p<.001  22q-dup < HC < 22q-del | F=1.40, df=2, p=.25 |
| Anterior limb of internal capsule right | F=18.38, df=2, p<.001  22q-dup < HC < 22q-del | F=1.71, df=2, p=.19 |
| Body of corpus callosum | F=6.44, df=2, p=.003  22q-dup < HC < 22q-del | F=27.19, df=2, p<.001  22q-del <HC <22q-dup |
| Cingulate gyrus left | F=10.14, df=2, p<.001  22q-dup < HC < 22q-del | F=21.33, df=2, p<.001  22q-del <HC <22q-dup |
| Cingulate gyrus right | F=3.29, df=2, p=.045  22q-dup < HC < 22q-del | F=21.93, df=2, p<.001  22q-del <HC <22q-dup |
| Cingulum hippocampus left | F=.83, df=2, p=.44 | F=12.21, df=2, p<.001  22q-del <HC <22q-dup |
| Cingulum hippocampus right | F=.20, df=2, p=.82 | F=15.82, df=2, p<.001  22q-del <HC <22q-dup |
| Cerebral peduncle left | F=9.80, df=2, p<.001  22q-dup < HC < 22q-del | F=.82, df=2, p=.45 |
| Cerebral peduncle right | F=11.46, df=2, p<.001  22q-dup < HC < 22q-del | F=.11, df=2, p=.89 |
| Corticospinal tract left | F=6.54, df=2, p=.003  22q-dup < HC < 22q-del | F=2.44, df=2, p=.097 |
| Corticospinal tract right | F=7.75, df=2, p<.001  22q-dup < HC < 22q-del | F=3.42, df=2, p=.040  HC <22q-del <22q-dup |
| External capsule left | F=1.23, df=2, p=.30 | F=18.14, df=2, p<.001  22q-del <HC <22q-dup |
| External capsule right | F=1.68, df=2, p=.20 | F=21.80, df=2, p<.001  22q-del <HC <22q-dup |
| Fornix | F=.56, df=2, p=.57 | F=4.49, df=2, p=.015  HC <22q-dup <22q-del |
| Genu of corpus callosum | F=7.15, df=2, p=.002  22q-dup < HC < 22q-del | F=16.28, df=2, p<.001  22q-del <HC <22q-dup |
| Inferior cerebellar peduncle left | F=3.88, df=2, p=.026  22q-dup < HC < 22q-del | F=5.30, df=2, p=.008  22q-del <HC <22q-dup |
| Inferior cerebral peduncle right | F=2.46, df=2, p=.095 | F=3.96, df=2, p=.025  22q-del <22q-dup <HC |
| Inferior fronto-occipital fasciculus left | F=3.04, df=2, p=.055 | F=6.12, df=2, p=.004  22q-del <HC <22q-dup |
| Inferior fronto-occipital fasciculus right | F=3.55, df=2, p=.035  22q-dup < HC < 22q-del | F=23.18, df=2, p<.001  22q-del <HC <22q-dup |
| Medial lemniscus left | F=4.34, df=2, p=.018  22q-dup< 22q-del< HC | F=.083, df=2, p=.92 |
| Medial lemniscus right | F=3.89, df=2, p=.026  22q-dup< 22q-del< HC | F=1.20, df=2, p=.31 |
| Posterior corona radiata left | F=2.75, df=2, p=.072 | F=29.46, df=2, p<.001  22q-del <HC <22q-dup |
| Posterior corona radiata right | F=4.02, df=2, p=.023  22q-dup < HC < 22q-del | F=32.97, df=2, p<.001  22q-del <HC <22q-dup |
| Posterior limb of internal capsule left | F=24.15, df=2, p<.001  22q-dup < HC < 22q-del | F=1.56, df=2, p=.22 |
| Posterior limb of internal capsule right | F=27.71, df=2, p<.001  22q-dup < HC < 22q-del | F=4.13, df=2, p=.021  22q-del <HC <22q-dup |
| Posterior thalamic radiation left | F=.48, df=2, p=.62 | F=31.90, df=2, p<.001  22q-del <HC <22q-dup |
| Posterior thalamic radiation right | F=5.15, df=2, p=.009  22q-dup< 22q-del< HC | F=35.24, df=2, p<.001  22q-del <HC <22q-dup |
| Retrolenticular part of internal capsule left | F=3.28, df=2, p=.045  22q-dup< 22q-del< HC | F=31.87, df=2, p<.001  22q-del <HC <22q-dup |
| Retrolenticular part of internal capsule right | F=3.34, df=2, p=.043  22q-dup < HC < 22q-del | F=25.03, df=2, p<.001  22q-del <HC <22q-dup |
| Splenium of corpus callosum | F=1.57, df=, p=.22 | F=28.98, df=2, p<.001  22q-del <HC <22q-dup |
| Superior cerebellar peduncle left | F=9.35, df=2, p<.001  22q-dup < HC < 22q-del | F=3.41, df=2, p=.040  22q-del <HC <22q-dup |
| Superior cerebellar peduncle right | F=7.24, df=2, p=.002  22q-dup < HC < 22q-del | F=5.78, df=2, p=.005  22q-del <HC <22q-dup |
| Superior corona radiata left | F=11.97, df=2, p<.001  22q-dup < HC < 22q-del | F=5.66, df=2, p=.006  HC< 22q-del <22q-dup |
| Superior corona radiata right | F=14.80, df=2, p<.001  22q-dup < HC < 22q-del | F=1.99, df=2, p=.15 |
| Superior fronto-occipital fasciculus left | F=4.47, df=2, p=.016  22q-dup< 22q-del< HC | F=.79, df=2, p=.46 |
| Superior fronto-occipital fasciculus right | F=3.83, df=2, p=.027  22q-dup< 22q-del< HC | F=1.08, df=2, p=.35 |
| Superior longitudinal fasciculus left | F=7.49, df=2, p<.001  22q-dup < HC < 22q-del | F=8.68, df=2, p<.001  HC <22q-del <22q-dup |
| Superior longitudinal fasciculus right | F=10.21. df=2, p<.001  22q-dup < HC < 22q-del | F=10.00, df=2, p<.001  HC <22q-del <22q-dup |
| Sagittal stratum left | F=1.26, df=2, p=.29 | F=28.89, df=2, p<.001  22q-del <HC <22q-dup |
| Sagittal stratum right | F=.31, df=2, p=.74 | F=41.17, df=2, p<.001  22q-del <HC <22q-dup |
| Uncinate fasciculus left | F=15.74, df=2, p<.001  2q-dup < HC < 22q-del | F=26.92, df=2, p<.001  22q-del <HC <22q-dup |
| Uncinate fasciculus right | F=89.28, df=2, p<.001  2q-dup < HC < 22q-del | F=24.34, df=2, p<.001  22q-del <HC <22q-dup |
| p<.001  p<.01  p<.05  Abbreviations: FAт = fractional anisotropy within tissue, FW = free-water | | |
